# Sonoelastography for differential diagnosis between malignant and benign parotid lesions: a meta-analysis

**DOI:** 10.1007/s00330-018-5609-6

**Published:** 2018-07-10

**Authors:** Yun-Fei Zhang, Hong Li, Xue-Mei Wang, Yun-Fei Cai

**Affiliations:** 1 Department of Ultrasound, The First Hospital of China Medical University, No. 155 Nanjing North Street, Heping District, Shenyang City, 110001 China; 2 Department of Transplantation and General Surgery, The First Hospital of China Medical University, No. 155 Nanjing North Street, Heping District, Shenyang City, 110001 China; 3 Department of Dermatology, The First Hospital of China Medical University, No. 155 Nanjing North Street, Heping District, Shenyang City, 110001 China

**Keywords:** Parotid neoplasms, Elasticity imaging techniques, Ultrasonography

## Abstract

**Objectives:**

To assess the performance of sonoelastography for differential diagnosis between malignant and benign parotid lesions using a meta-analysis.

**Methods:**

An independent literature search of English medical databases, such as PubMed, Embase and Medline (Embase.com), Web of Science, Cochrane Library and Ovid was performed. The diagnostic accuracy of sonoelastography was compared with that of histopathology and/or cytology, which was used as reference standard. The pooled sensitivity, specificity, diagnostic odds ratio (DOR) and area under the curve (AUC) were calculated to evaluate the accuracy of sonoelastography. A meta-regression analysis evaluating imaging mechanisms, shear wave elastography techniques, assessment methods and QUADAS scores was performed.

**Results:**

Ten eligible studies that included a total sample of 711 patients with 725 parotid lesions were included. Sonoelastography showed a pooled sensitivity of 0.67 (95% CI 0.59–0.74), specificity of 0.64 (95% CI 0.60–0.68), DOR of 8.00 (95% CI 2.96–21.63) and an AUC of 0.77. The results of the meta-regression analysis revealed that no heterogeneity was due to the imaging mechanism (*p =* 0.119), shear wave elastography technique (*p =* 0.473) or QUADAS score (*p =* 0.462). However, the assessment method was a significant factor that affected the study heterogeneity (*p =* 0.035). According to the subgroup analysis, quantitative and semiquantitative methods performed better than qualitative ones.

**Conclusion:**

Overall, sonoelastography has a limited value for differential diagnosis between malignant and benign parotid lesions. Quantitative and semiquantitative methods perform better than qualitative ones.

**Key Points:**

*• Overall, sonoelastography has a limited value for differential diagnosis between malignant and benign parotid lesions.*

*• Quantitative and semiquantitative assessment methods perform better than qualitative ones.*

*• Semiquantitative and quantitative methods are automatically calculated by an ultrasound machine and are thus less operator-dependent.*

## Introduction

Ultrasonography, CT and MRI are non-invasive imaging methods that are commonly used for the evaluation of parotid tumours. However, these conventional imaging methods are less accurate owing to the overlap in the appearance of parotid tumours. Some malignancies that contain a large amount of serous and mucoid contents are well defined with a homogeneous appearance and resemble benign lesions. In addition, haemorrhage and calcification in benign tumours may result in a heterogeneous appearance that resembles a malignancy [1–5]. Although ultrasound-guided fine-needle aspiration cytology (FNAC) is considered the gold standard for preoperative diagnosis [6], it is an invasive method and, as a general rule, non-invasive methods are preferred when the results are similar [7].

Sonoelastography is an innovative diagnostic imaging tool that assesses tissue stiffness [8]. Since malignant tissues are generally stiffer than benign components, sonoelastography has been used in many organs, such as the breast, thyroid and prostate, for differential diagnosis between malignant and benign lesions [9–14]. Recently, numerous studies have been published on the role of sonoelastography for differentiating between malignant and benign parotid lesions. However, there are large differences in the results, with a sensitivity ranging from 40% to 100% and a specificity ranging from 26% to 97% [7, 8, 15–20]. Therefore, this study aimed to assess the performance of sonoelastography for differential diagnosis between malignant and benign parotid lesions using a meta-analysis.

## Materials and methods

### Literature search

The study complied with the PRISMA recommendations [21, 22]. An independent literature search of English medical databases including PubMed, Embase and Medline (Embase.com), Web of Science, Cochrane Library and Ovid was performed to identify all studies evaluating differential diagnosis between malignant and benign parotid lesions. The strategies are shown in Table 1. Duplicated articles were excluded manually. Unpublished relative data were considered as well, but no suitable studies were identified for inclusion. The study was performed by two independent researchers. This literature search was updated until 30 October 2017 and a beginning date limit was not used.

**Table 1:** Search strategy of each database

Database	Strategy
PubMed	(((((((("Parotid Neoplasms"[Mesh]) OR parotid neoplasm) OR parotid cancer) OR parotid carcinoma) OR parotid tumor) OR parotid mass) OR parotid lesion)) AND (((((((("Elasticity Imaging Techniques"[Mesh]) OR elasticity imaging technique) OR tissue elasticity imaging) OR elastography) OR vibro acoustography) OR acoustic radiation force impulse) OR sonoelastography) OR elastogram)
Embase and Medline (Embase.com)	(#1) parotid AND neoplasm OR (parotid AND cancer) OR (parotid AND carcinoma) OR ( parotid AND tumor) OR (parotid AND mass) OR (parotid AND lesion) (#2) elasticity AND imaging AND technique OR (tissue AND elasticity AND imaging) OR elastography OR (vibro AND acoustography) OR (acoustic AND radiation AND force AND impulse) OR sonoelastography OR elastogram (#3) #1 AND #2
Cochrane Library	(#1) Mesh descriptor: [Parotid Neoplasms] explode all trees (#2) parotid neoplasm OR parotid cancer OR parotid carcinoma OR parotid tumor OR parotid mass OR parotid lesion (Word variations have been searched) (#3) #1 OR #2 (#4) Mesh descriptor: [Elasticity Imaging Techniques] explode all trees (#5) elasticity imaging technique OR tissue elasticity imaging OR elastography OR vibro acoustography OR acoustic radiation force impulse OR sonoelastography OR elastogram (Word variations have been searched) (#6) #4 OR #5 (#7) #3 AND #6
Web of Science	TOPIC: ((parotid neoplasm) OR (parotid cancer) OR (parotid carcinoma) OR (parotid tumor) OR (parotid mass) OR (parotid lesion)) AND TOPIC: ((elasticity imaging technique) OR (tissue elasticity imaging) OR (elastography) OR (vibro acoustography) OR (acoustic radiation force impulse) OR (sonoelastography) OR (elastogram))
OVID	(#1) (parotid neoplasm OR parotid cancer OR parotid carcinoma OR parotid tumor OR parotid mass OR parotid lesion).af. (#2) (elasticity imaging technique OR tissue elasticity imaging OR elastography OR vibro acoustography OR acoustic radiation force impulse OR sonoelastography OR elastogram).af. (#3) #1 AND #2

### Inclusion and exclusion criteria

All the articles were assessed independently by two researchers. The inclusion criteria for the studies were as follows: (1) The study was approved by an ethics committee or institutional review board. (2) The diagnostic performance of sonoelastography for the differential diagnosis between malignant and benign parotid lesions was evaluated in the study. (3) Postoperative pathology and/or fine-needle aspiration cytology (and/or histology) results were used as the reference standard in the study. (4) Complete reported data were available to calculate the true positive (TP), false positive (FP), false negative (FN) and true negative (TN) cases. The exclusion criteria for the studies were as follows: (1) Reviews, case reports, letters, conference reports, editorial comments and articles that were not published in English were excluded. (2) In studies with insufficient data, the corresponding authors were contacted and requested to provide the missing data via e-mail. The studies were excluded if the author did not reply within 15 days. (3) When two or more studies were performed by the same department, the study that was older or that had the smaller number of patient samples was excluded. All the disagreements were resolved by consensus.

### Data extraction

Two investigators extracted the data independently. All relevant data including first author, country where the study was performed, published year, patient age, proportion of male and female patients, number of patients, number of lesions, reference standard, type of lesions, ultrasound system, sonoelastography index, cut-off value and number of TPs, FPs, FNs and TNs were extracted. The cut-off value was defined according to the Youden method if it was not clearly provided by the author. Disagreements were resolved by consensus.

### Quality assessment

The methodological qualities of primary studies were assessed with the Quality Assessment of Diagnostic Accuracy Studies (QUADAS) criteria [23]. The defined questions were answered as yes, no or unclear, and ultimately, a maximum score of 14 was used to estimate the quality of each article. Two researchers completed all the items and disagreements were resolved by consensus.

### Data analysis

The statistical software Meta-Disc (Version 1.4, Unit of Clinical Biostatistics team of the Ramón y Cajal Hospital), STATA (Version 12.0, Stata Corporation) and SPSS Statistics (Version 17.0, SPSS Inc.) were used in this study. The Spearman correlation coefficient was used to analyse the threshold effect. The heterogeneity was evaluated by the Cochran *Q* statistic and the *I*^2^ test. A random effects model was used when the *p* value of heterogeneity was less than 0.05 or the *I*^2^ was at least 50%, otherwise a fixed effects model was used. The pooled sensitivity, specificity, diagnostic odds ratio (DOR), area under the curve (AUC) and Q* index were calculated using Meta-Disc. Potential sources of heterogeneity were explored with a meta-regression analysis. Deeks’ funnel plot was generated in STATA to analyse the potential publication bias, with a *p <* 0.05 indicating potential publication bias. Interobserver agreement was analysed with Cohen’s κ analysis using SPSS software while screening articles and applying the QUADAS criteria.

## Results

### Literature search and characteristics of included studies

**Fig. 1:**
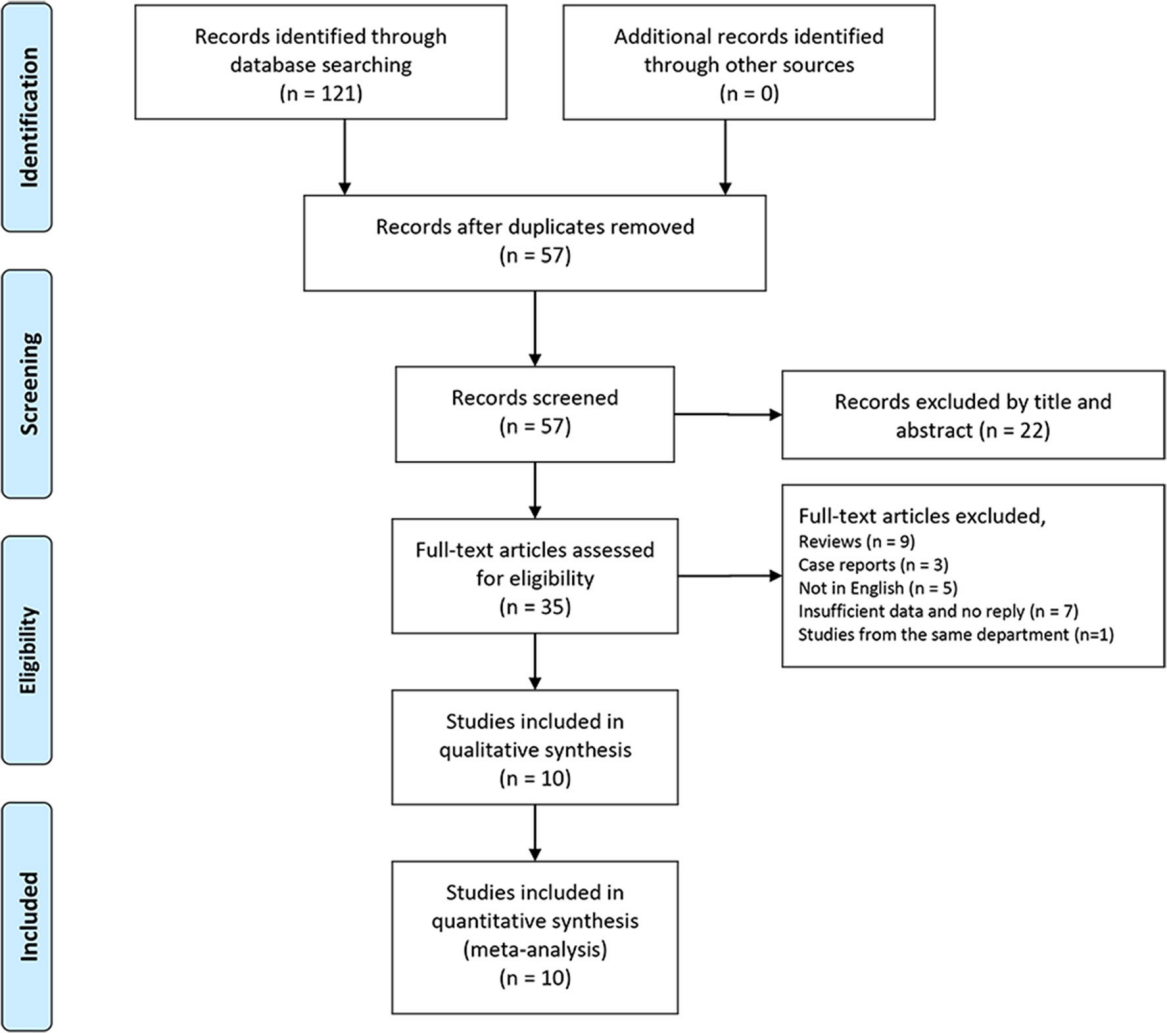
Flow diagram of study selection. *n* = number of studies

Ten relative studies including 711 patients with 725 parotid lesions were included in the meta-analysis after literature search, which were published from 2012 to 2017 [6–8, 15–20, 24] (Fig. 1). The main characteristics of the included studies are summarised in Table 2. Controversies occurred between two observers in the step when the records were excluded by title and abstract. However, it showed an excellent interobserver agreement (κ = 0.86; 95% CI 0.72–0.99). Ultimately, all the controversial articles were included in this step. There was no disagreement in other steps of screening (κ = 1).

**Table 2:** Main characteristics of included studies

	Author	Country	Year	Age (avg. or median)	Male/female	Number of patients	Number of lesions	Reference standard	Type of lesions (number of lesions)	Ultrasound system	Index of elastography	Cut-off value	TP	FP	FN	TN
1	Klintworth et al [8]	Germany	2012	53.3	27/30	57	57	Postoperative pathology	Squamous cell carcinoma (3), mucoepidermoid carcinoma (1), salivary duct carcinoma (1), basal cell carcinoma (1), carcinoma ex pleomorphic adenoma (1), metastatic malignant melanoma (1); pleomorphic adenoma (22), Warthin tumour (20), cystadenoma (2), lipoma (2), cyst (3)	Siemens Ltd., Healthcare Sector, Acuson S2000	Garland sign	Garland sign (+)	3	2	5	47
2	Yerli et al [24]^*^	Turkey	2012	47.4	15/15	30	30	Postoperative pathology (*n* = 33) or clinical indications, fine-needle aspiration biopsy (*n* = 3)	Adenocarcinomas (2), mucoepidermoid carcinomas (2), adenoid cystic carcinomas (1), malignant lymphoma (1); Warthin tumours (10), pleomorphic adenomas (10), lymphadenopathies (3), abscesses (1)	Hitachi Medical Systems, EUB-7000	ES (4-point)	ES3	4	8	2	16
3	Badea et al [16]	Romania	2013	NA	15/5	20	20	postoperative pathology	Parotid carcinomas (6), metastases (2); all pleomorphic adenomas (12)	General Electric GE 7, GE 8, GE 9; Phillips, iU22; Siemens S 2000	Tactile Elastography or ARFI	NA	8	6	0	6
4	Celebi and Mahmutoglu [17]	Turkey	2013	47.19	36/39	75	81	Postoperative pathology (*n* = 73), FNAC (*n* = 6), both (*n* = 2)	Lymphoma (9), mucoepidermoid carcinoma (6), adenoid cystic carcinoma (5), metastases (3), myoepithelial malignant tumour (1), pleomorphic adenocarcinoma (2), salivary duct carcinoma (2), acinic cell carcinoma (2) and basal cell carcinoma (2); pleomorphic adenoma (28), Warthin tumour (10), lymphadenopathy (9), cystic adenoma/infected cyst rupture (2)	Siemens Medical Solutions, Siemens S2000	CSES (4-point)	CSES3	19	19	13	30
5	Wierzbicka et al [19]	Poland	2013	54	27/16	43	43	Postoperative pathology	Adenocarcinoma (3), malignant lymphoma (2), clear cell carcinoma (1), non-differentiated cancer (1), squamous cell cancer (1), cancer from pleomorphic adenoma (1) and salivary duct cancer (1); pleomorphic adenoma (23), Warthin tumour (5), monomorphic adenoma (2), neurofibroma (1), cyst (1), basal cell adenoma (1)	Supersonic Imagine, Aixplorer	ES (5-point)	ES4	4	1	6	32
6	Yu et al [20]	China	2016	45	NA	51	51	Postoperative pathology	Mucoepidermoid carcinomas (5), acinic cell carcinomas (3), salivary duct carcinomas (3), basal cell carcinomas (2), adenoid cystic carcinoma (1), adenocarcinoma (1), carcinoma ex pleomorphic adenoma (1); pleomorphic adenoma (16), adenolymphomas (15), basal cell adenomas (2), multiple nodular acidophil adenoma (1), myoepithelioma (1)	Siemens Medical Solutions, Acuson S2000	SWV	2.76 m/s	11	1	5	34
7	Altinbas et al [15]	Turkey	2017	60.01	26/20	46	54	Fine-needle aspiration biopsy (cytological and histological results)	Malignant melanoma (3), salivary duct carcinoma (2), squamous cell carcinoma (1), adenoid cystic carcinoma (1), low-grade adenocarcinoma (1), mucoepidermoid carcinoma (1), Merkel cell carcinoma (1); Warthin tumour (18), pleomorphic adenoma (8), reactive lymphoid hyperplasia (6), lipoma (3), granulomatous inflammation (2), basal cell adenoma (2), lymphoepithelial cyst (1), sialadenitis and abscess (1), sialadenosis (1), primary benign oncocytic neoplasm (1), Rosai-Dorfman disease (1)	GE HealthCare, Logiq S7 Expert	E-index	3	7	15	3	29
8	Cantisani et al [7]	Italy	2017	56	NA	63	63	Postoperative pathology and FNAC	Mucoepidermoid carcinoma (5), malignant lymphoma (3), squamous cell carcinoma (2), acinic cell carcinoma (2), salivary duct carcinoma (2) and squamous cell carcinoma metastasis (2); pleomorphic adenoma (18), Warthin tumour (17), oncocytoma (5), lymphoepithelial cyst (3), ductal cyst (2), benign vascular tumour (2).	Samsung Medison, Accuvix A30, RS 80 A	ECI	3.5	15	5	1	42
9	Herman et al [6]^*^	Czech Republic	2017	60	66/58	124	124	Postoperative pathology	Squamous cell carcinoma (8), low grade salivary tumour (6), high grade salivary tumour (7), lymphoma (3), melanoma (2), sarcoma (1), neuroendocrine carcinoma (1); pleomorphic adenoma (49), Warthin tumour (33), other benign lesions (14) including oncocytic adenomas, lipomas, lipomatosis, basal cell adenoma, non-sebaceous lymphadenoma, branchiogenic cyst, and chronic inflammation	SuperSonic Imagine,Aixplorer	CSV	1025	18	17	10	79
10	Mansour et al [18]^#^	Germany	2017	58.6	NA	202	202	Postoperative pathology	Primary parotid carcinomas (10), secondary parotid carcinomas (13), non-Hodgkin lymphomas (9); pleomorphic adenomas (64), Warthin tumours (73), basal cell adenomas (6), oncocytomas (3), polymorphic adenomas (2), myoepithelioma (1), sebaceous lymphadenoma (1), ductal dilatations (8), lymphoepithelial cysts (5), cystadenomas (4), chronic inflammations (3)	Siemens Healthcare, Acuson S2000	ES (3-point)	ES2	22	126	10	44

*Avg.* average, *FNAC* fine needle aspiration cytology, *SWV* shear wave velocity, *CSV* coefficient of stiffness variability (maximum of stiffness/minimum of stiffness), *CSES* consensus sonoelastography scores, *ES* elastographic scoring, *ECI* elasticity contrast index, *ARFI* acoustic radiation force impulse, *TP* true positive, *FN* false negative, *FP* false positive, *TN* true negative, *NA* not available

*Missing data provided by corresponding author via e-mail

^#^Another study [25] from the same department was omitted

### Quality assessment

Quality assessment of each study is shown in Table 3. Most of the indexes were adequate and resulted in a high QUADAS score. However, it was unclear if the pathologist was blinded to the sonoelastography results in all the studies. In one study, only pleomorphic adenomas were identified in the benign group [16]. In one study, it was unclear if the radiologist was blinded to the pathology [20], and in another study, the ultrasound examiners were aware of the histological properties of the respective lesions [8]. The interobserver agreement was good (κ = 0.77; 95% CI 0.60–0.93).

**Table 3:** Quality assessment of the included studies using the “QUADAS” questionnaire

QUADAS questionnaire	Klintworth et al 2012 [8]	Yerli et al 2012 [24]	Badea et al 2013 [16]	Celebi and Mahmutoglu 2013 [17]	Wierzbicka et al 2013 [19]	Yu et al 2016 [20]	Altinbas et al 2017 [15]	Cantisani et al 2017 [7]	Herman et al 2017 [6]	Mansour et al 2017 [18]
1: Was the spectrum of patient representative of the patients who will receive the test in practice?	Yes	Yes	No	Yes	Yes	Yes	Yes	Yes	Yes	Yes
2: Were selection criteria clearly described?	Yes	Yes	Yes	Yes	Yes	Yes	Yes	Yes	Yes	Yes
3: Is the reference standard likely to correctly classify the target condition?	Yes	Yes	Yes	Yes	Yes	Yes	Yes	Yes	Yes	Yes
4: Is the time period between reference standard and index test short enough to be sure that the target condition did not change between the two tests?	Yes	Yes	Yes	Yes	Yes	Yes	Yes	Yes	Yes	Yes
5: Did the whole sample, or a random selection of the sample, receive verification using a reference standard of diagnosis?	Yes	Yes	Yes	Yes	Yes	Yes	Yes	Yes	Yes	Yes
6: Did patients receive the same reference standard regardless of the index test result?	Yes	Yes	Yes	Yes	Yes	Yes	Yes	Yes	Yes	Yes
7: Was the reference standard independent of the index test (i.e., the index test did not form part of the reference standard)?	Yes	Yes	Yes	Yes	Yes	Yes	Yes	Yes	Yes	Yes
8: Was the execution of the index test described in sufficient detail to permit replication of the test?	Yes	Yes	Yes	Yes	Yes	Yes	Yes	Yes	Yes	Yes
9: Was the execution of the reference standard described in sufficient detail to permit replication?	Yes	Yes	Yes	Yes	Yes	Yes	Yes	Yes	Yes	Yes
10: Were the index test results interpreted without knowledge of the results of the reference standard?	No	Yes	Yes	Yes	Yes	Unclear	Yes	Yes	Yes	Yes
11: Were the reference standard results interpreted without knowledge of the results of the index test?	Unclear	Unclear	Unclear	Unclear	Unclear	Unclear	Unclear	Unclear	Unclear	Unclear
12: Were the same clinical data available when test results were interpreted as would be available when the test is used in practice?	Yes	Yes	Yes	Yes	Yes	Yes	Yes	Yes	Yes	Yes
13: Were un-interpretable/intermediate test results reported?	Yes	Yes	Yes	Yes	Yes	Yes	Yes	Yes	Yes	Yes
14: Were withdrawals from the study explained?	Yes	Yes	Yes	Yes	Yes	Yes	Yes	Yes	Yes	Yes
QUADAS score	12.5	13.5	12.5	13.5	13.5	13	13.5	13.5	13.5	13.5

### Diagnostic accuracy for differential diagnosis between malignant and benign parotid lesions

**Fig. 2:**
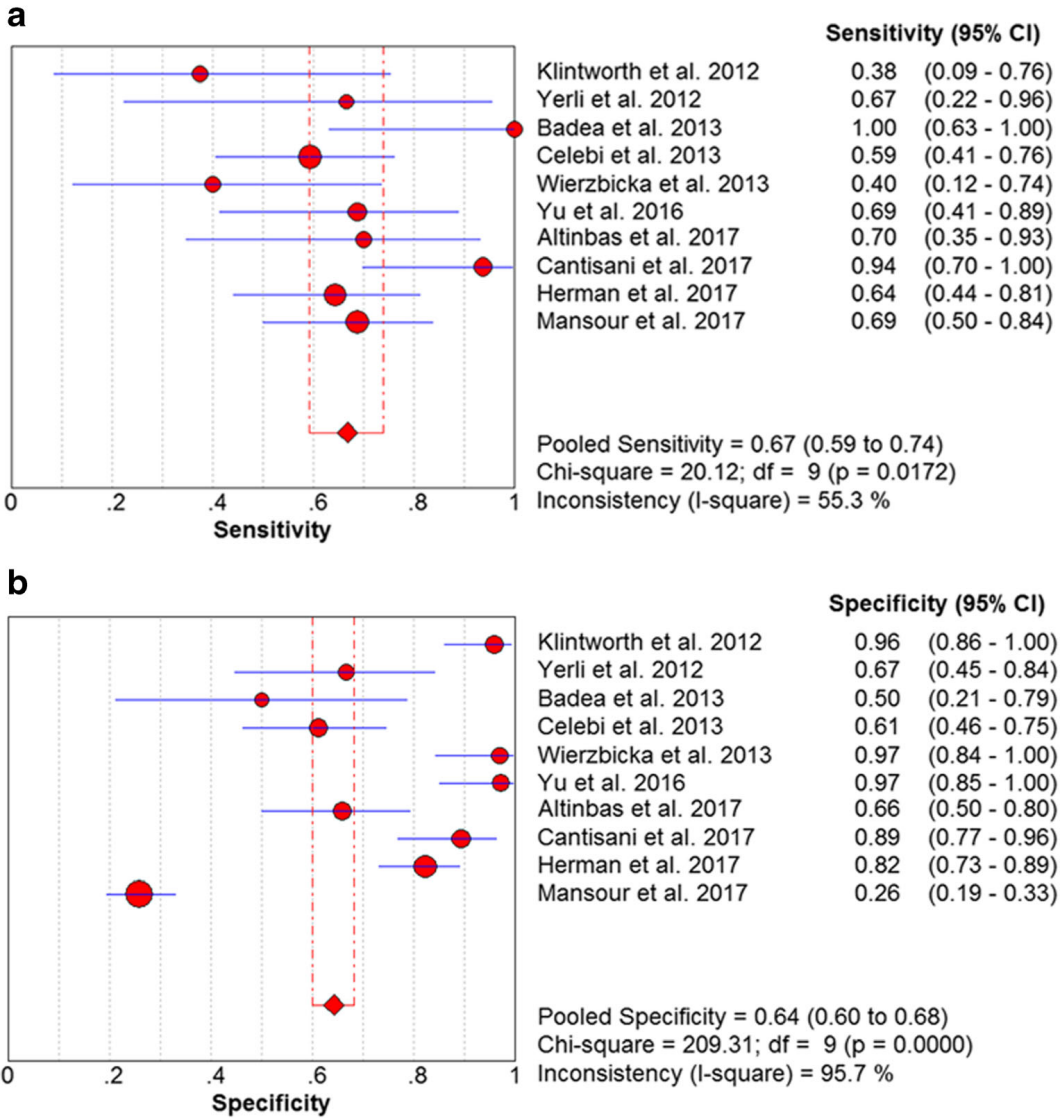
Forest plots of the pooled sensitivity (**a**) and specificity (**b**) of sonoelastography for differentiating between malignant and benign parotid lesions

**Fig. 3:**
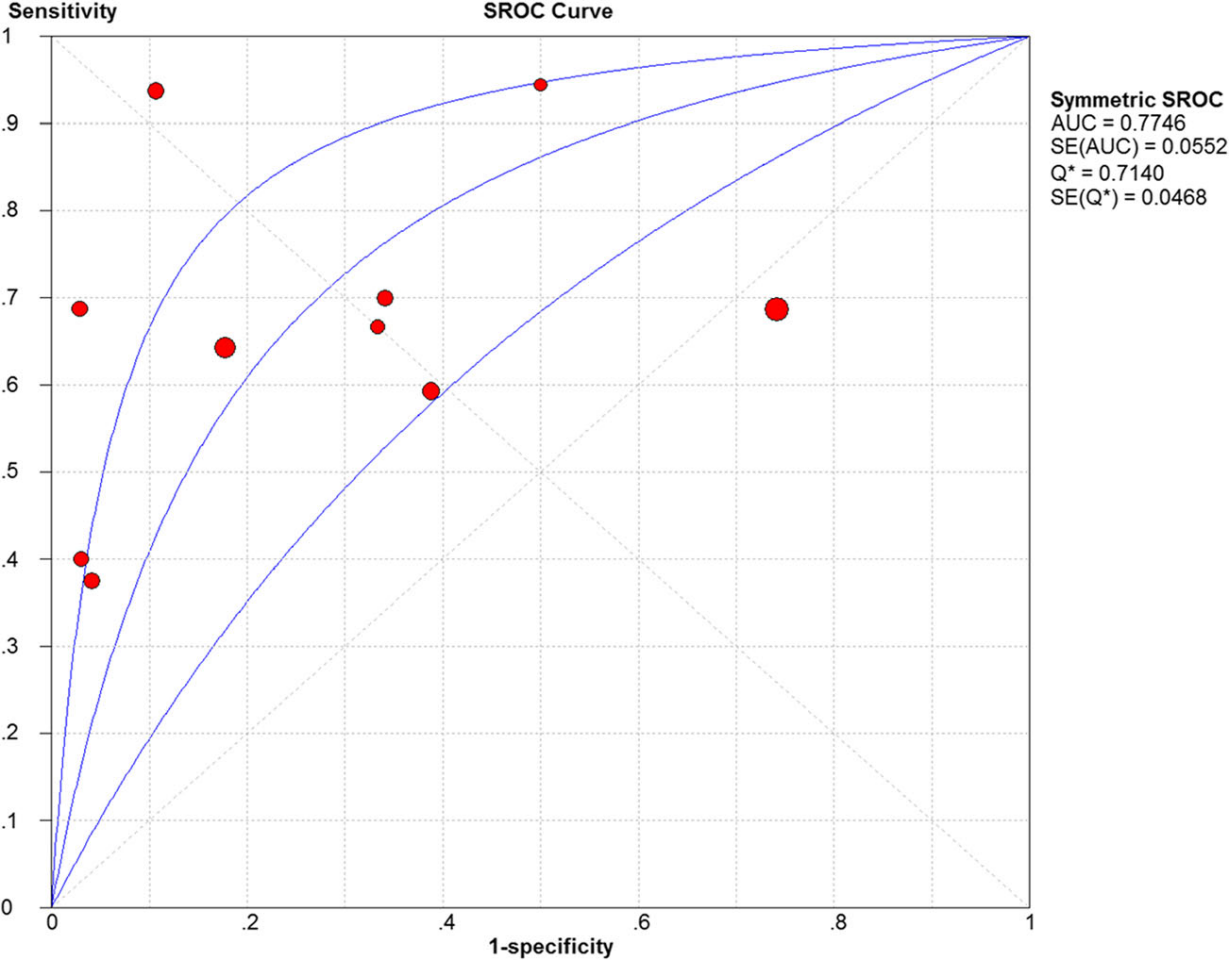
Summary receiver operating characteristic (SROC) curve on sonoelastography for differentiating between malignant and benign parotid lesions. The middle curve is the SROC curve. The upper and lower curves show the 95% confidence intervals

No heterogeneity was identified by analysis of the diagnostic threshold, with a Spearman correlation coefficient of 0.389 (*p =* 0.266). The diagnostic accuracy of sonoelastography for differential diagnosis between malignant and benign parotid lesions was computed on the basis of a pooled sensitivity of 0.67 (95% CI 0.59–0.74), specificity of 0.64 (95% CI 0.60–0.68) and DOR of 8.00 (95% CI 2.96–21.63) (Fig. 2). An overall moderate degree of accuracy was identified by the summary receiver operating characteristic (SROC) curve with an AUC of 0.77 (Q* = 0.71) (Fig. 3).

### Heterogeneity results

The Cochran *Q* test and the *I*^2^ test revealed significant heterogeneity with *p* < 0.001 and *I*^2^ = 77.2%. To further explore the sources of heterogeneity, a meta-regression analysis evaluating imaging mechanisms (group 1, strain elastography (SE); group 2, shear wave elastography (SWE)), shear wave elastography techniques (group 1, supersonic shear imaging (SSI) with a SuperSonic Imagine Aixplorer; group 2, acoustic radiation force impulse imaging (ARFI) with a Siemens S2000), assessment methods (group 1, qualitative; group 2, quantitative or semiquantitative) and QUADAS scores was performed. The results indicated that no heterogeneity was due to the imaging mechanism (*p =* 0.119), shear wave elastography technique (*p =* 0.473) or QUADAS score (*p =* 0.462). However, the assessment method was a significant factor that affected the study heterogeneity (*p =* 0.035). Compared with qualitative assessment methods, quantitative and semiquantitative methods performed better (Table 4).

**Table 4:** Results of the meta-regression and subgroup analysis for differential diagnosis between malignant and benign parotid lesions

Subgroup	Number of studies	Pooled sensitivity (95% CI)	Pooled specificity (95% CI)	Pooled DOR (95% CI)	AUC	*p* value
Mechanism						0.119
SWE	6	0.61 (0.52–0.70)	0.62 (0.57–0.66)	6.39 (1.82–22.35)	0.67	
SE	3	0.81 (0.64–0.93)	0.76 (0.67–0.83)	11.67 (1.56–87.41)	0.64	
SWE technique						0.473
ARFI	4	0.63 (0.52–0.73)	0.51 (0.45–0.57)	4.91 (0.96–25.05)	0.65	
SSI	2	0.58 (0.41–0.74)	0.86 (0.79–0.92)	9.50 (3.99–22.63)	NA	
Assessment method						0.035*
Qualitative	5	0.59 (0.48–0.69)	0.52 (0.46–0.58)	3.38 (1.08–10.57)	0.63	
Qualitative or semiquantitative	4	0.73 (0.61–0.83)	0.83 (0.77–0.88)	18.64 (4.51–77.07)	0.88	
QUADAS score						0.462
13.5	7	0.66 (0.58–0.74)	0.59 (0.54–0.63)	5.41 (1.81–16.13)	0.74	
≤ 13	3	0.69 (0.50–0.84)	0.91 (0.83–0.96)	26.56 (6.91–102.11)	0.90	

*SE* strain elastography, *SWE* shear wave elastography, *SSI* supersonic shear imaging, *ARFI* acoustic radiation force impulse imaging, *NA* not available

*Meta-regression, *p* < 0.05

### Evaluation of publication bias

**Fig. 4:**
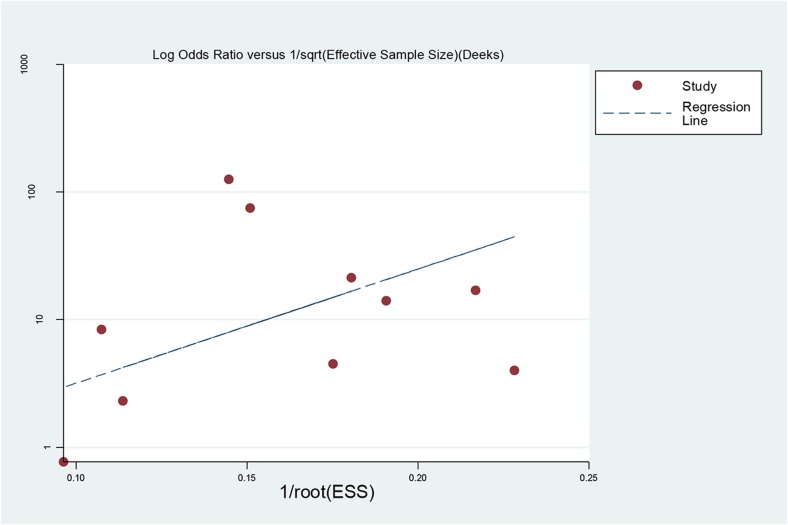
Funnel plot for evaluating potential publication bias. Each solid circle represents a study in the meta-analysis. The line is the regression line

Publication bias was explored with a Deeks’ funnel plot and no significant differences were detected in this meta-analysis (*p =* 0.143) (Fig. 4).

## Discussion

Our current meta-analysis demonstrated that sonoelastography showed a pooled sensitivity of 0.67 (95% CI 0.59–0.74) and specificity of 0.64 (95% CI 0.60–0.68) for differential diagnosis between malignant and benign parotid lesions. The pooled DOR was 8.00 (95% CI 2.96–21.63) and the AUC was 0.77. The meta-regression analysis results revealed that the assessment method was a significant factor affecting study heterogeneity (*p =* 0.035). However, the summary estimates did not differ between SE and SWE (*p* = 0.119) or between ARFI and SSI (*p* = 0.473).

Recently, several original studies have focused on the value of sonoelastography for differentiating between malignant and benign parotid lesions. Sonoelastography is a novel ultrasonographic technique for assessing tissue elasticity and stiffness. Theoretically, malignant parotid tumours should be stiffer than benign ones. However, the situation seems complicated. Some authors have described the great performance of sonoelastography for differentiating between malignant and benign lesions with a high sensitivity of 94% and a specificity of 89% [7]. Some have described a relatively lower but still clear performance of sonoelastography, with a sensitivity of 70% and specificity of 66% [15]. However, others have described that there was no benefit of sonoelastography for differentiating between malignant and benign tumours; only cystic lesions or cystic areas within a lesion were reliably identified [18]. Our meta-analysis ultimately revealed a pooled sensitivity of 67% and a pooled specificity of 64% for differentiating between malignant and benign parotid lesions. Therefore, we believe that the overall value of sonoelastography for differential diagnosis was limited and not satisfactory.

Heterogeneity was revealed in our study. Therefore, a meta-regression analysis was performed to further explore the potential sources. The results showed that there was no difference between SE and SWE or between ARFI and SSI. However, the assessment method was a significant factor affecting study heterogeneity. Quantitative and semiquantitative methods performed better than qualitative ones. In this subgroup, there was a higher pooled sensitivity of 0.73, specificity of 0.83, DOR of 18.64 and an AUC of 0.88. This was probably because qualitative methods were usually performed with a scoring system that was subjectively used by operators and was thus more operator-dependent. However, semiquantitative and quantitative methods were automatically calculated by an ultrasound machine and were thus less operator-dependent.

Another potential source of heterogeneity might be the histopathological variety in malignant and benign parotid lesions. Celebi and Mahmutoglu [17] indicated that the diagnostic value of sonoelastography for evaluating pleomorphic adenomas, Warthin tumours, adenoid cystic carcinoma and high-grade tumours was low, whereas the diagnostic rates for low-grade tumours, such as mucoepidermoid carcinoma, acinic cell carcinoma and metastases of basal cell carcinoma, were better. Pleomorphic adenomas contained variable proportions of chondroid and/or myxoid matrix, which contained different amounts of fluid. Warthin tumours contained different amounts of lymphatic, cellular, mucous and fluid components. Thus, the two types of benign tumours could be solid, solid and cystic, or completely cystic, which resulted in a wide variety in stiffness. In a small sample study of 20 patients with only pleomorphic adenomas included in the benign group, 50% (6/12) of the adenomas were misdiagnosed as malignancies [16]. We tried to analyse whether sonoelastography could differentiate between low-grade parotid tumours and high-grade and benign ones. We also tried to analyse the effects of the different components in pleomorphic adenomas and Warthin tumours on sonoelastography. However, both of these analyses were not accomplished because, in most of the studies, the data were not recorded.

A strict procedure was carried out to screen the articles and ultimately 10 relative studies were identified. Deeks’ funnel plots showed no significant publication bias. Most of the studies were high quality according to the QUADAS questionnaire. A meta-regression revealed that the QUADAS score was not a significant factor affecting study heterogeneity. However, the QUADAS score seemed to perform better in relatively lower quality studies, as shown in Table 4. In one study [20], it was unclear whether the observers knew the histopathological results before analysing the images. In another study, the observers were aware of histological properties before reviewing the images and videos [8]. These unblinded studies probably had better performance and influenced the results. In addition, in all the studies it was unclear whether the histopathology reviewer knew the results of sonoelastography evaluations, which probably caused heterogeneity and influenced the results as well. To the best of our knowledge, this is the first meta-analysis to assess the diagnostic value of sonoelastography merely for differentiating between malignant and benign parotid lesions, except for salivary gland masses [26].

There are some limitations in our study. First, relatively few studies were included (i.e. ten). Second, we failed to acquire unpublished data and language limitations might have affected the reliability of the results. Third, postoperative pathology was used as a reference standard for tumour detection in most of the studies in this meta-analysis; however, in one study [15], only cytological and histological results from ultrasound-guided fine needle aspiration biopsy were used as reference standards, and in another two studies, cytology results from ultrasound-guided fine needle aspiration were used in six cases [17] and two cases [24], respectively. Although cytology and histology of fine-needle aspiration biopsy are suggested diagnostic methods for most parotid tumours, these methods have variable success with sensitivity ranging from 57% to 98%, specificity ranging from 56% to 100% and accuracy ranging from 78% to 98% [7].

In conclusion, this meta-analysis shows that sonoelastography has a limited value for differential diagnosis between malignant and benign parotid lesions. Quantitative and semiquantitative methods performed better than qualitative ones. Further large-sample, prospective, multicentre studies evaluating these two assessment methods are needed to confirm the findings. In addition, more studies should focus on the correlation between sonoelastography and corresponding histopathological changes in the future.

## Abbreviations

DOR: Diagnostic odds ratio
FN: False negative
FNAC: Fine-needle aspiration cytology
FP: False positive
QUADAS: Quality Assessment of Diagnostic Accuracy Studies
SROC: Summary receiver operating characteristic
TN: True negative
TP: True positive
